# Activation of PXR by Alpinetin Contributes to Abrogate Chemically Induced Inflammatory Bowel Disease

**DOI:** 10.3389/fphar.2020.00474

**Published:** 2020-04-21

**Authors:** Zhilun Yu, Bei Yue, Lili Ding, Xiaoping Luo, Yijing Ren, Jingjing Zhang, Sridhar Mani, Zhengtao Wang, Wei Dou

**Affiliations:** ^1^ Shanghai Key Laboratory of Formulated Chinese Medicines, Institute of Chinese Materia Medica, Shanghai University of Traditional Chinese Medicine, Shanghai, China; ^2^ Departments of Medicine and Genetics, Albert Einstein College of Medicine, New York, NY, United States

**Keywords:** inflammatory bowel disease, pregnane X receptor, nuclear factor-kappa B, xenobiotics, alpinetin

## Abstract

Alpinetin is a naturally occurring flavonoid from the ginger plants. We previously reported the identification of alpinetin as a ligand of human pregnane X receptor (hPXR). The current study investigated the role of alpinetin as a putative PXR activator in ameliorating chemically induced inflammatory bowel disease (IBD). We found that oral administration of alpinetin significantly alleviated the severity of dextran sulfate sodium (DSS)-induced colitis in mice by decreasing the inflammatory infiltration, the levels of the pro-inflammatory mediators, and the PXR target genes in the colon. *In vitro*, alpinetin blocked the nuclear translocation of p-p65 in lipopolysaccharide (LPS)-stimulated RAW264.7 macrophages. Further, alpinetin significantly upregulated PXR target genes and inhibited TNF-α-induced NF-κB-luciferase activity in LS174T colorectal cells; however, this regulatory effects were lost when cellular PXR gene was knocked down. In PXR transactivation assays, alpinetin increased both mouse and human PXR transactivation in a dose-dependent manner. Ligand occluding mutants, S247W/C284W and S247W/C284W/S208W, in hPXR-reporter assays, abrogated alpinetin-induced hPXR transactivation. Finally, alpinetin bound to the hPXR-ligand-binding domain (LBD) was confirmed by competitive ligand binding assay. The current study significantly extends prior observations by validating a PXR/NF-κB regulatory mechanism governing alpinetin’s anti-inflammatory effects in a murine model of IBD.

## Introduction

Pregnane X receptor (PXR, NR1I2), known as a ligand-dependent transcription factor, is a member of the nuclear receptors superfamily and is primarily expressed in the liver, intestine, and kidney. Ligand-activated PXR heterodimerizes with retinoid X receptor alpha (RXRα) and binds to the DNA response elements on the promoters of target genes. As a xenobiotic sensor, PXR regulates the transcription of genes encoding drug-metabolizing enzymes and transporters involved in the clearance of xenobiotic chemicals and organisms from the body (Mani et al., 2013). PXR has a bulky and flexible ligand-binding pocket that enables it to bind a wide spectrum of structurally diverse endogenous and exogenous ligands, such as prescription drugs, dietary supplements, bacterial products, environmental pollutants, endogenous hormones, and bile acids (Cheng et al., 2012; Mani et al., 2013). Notably, the ligands of PXR display significant interspecies difference in PXR activation profiles. For example, known human (h) PXR ligand rifampicin is an efficacious activator of hPXR, as evidenced by increased cytochrome P450 (Cyp)3a4 gene expression, whereas pregnenolone 16α-carbonitrile (PCN) is a potent activator of mouse (m) PXR.

Besides its critical role in xenobiotic metabolism and detoxification, PXR exerts potential anti-inflammatory effect against inflammatory bowel disease (IBD). PXR knockout mice are much easier to develop colitis than wild-type mice (Shah et al., 2007). The expression levels of PXR and its target genes are reduced in the intestinal samples from IBD patients compared with the normal individuals (Langmann et al., 2004). Single-nucleotide polymorphisms in the PXR gene have been linked to a reduction in PXR expression and an increase in the IBD susceptibility in humans (Dring et al., 2006). In addition, there is a reciprocal suppression between PXR and nuclear factor-kappa B (NF-κB) signaling pathway. PXR activation is known to suppress the activity of NF-κB and the expression of NF-κB target genes, while activation of NF-κB also represses PXR activation and the expression of PXR target cytochromes (CYPs) genes (Zhou et al., 2006). In PXR knockout mice, the expression of NF-κB target genes is upregulated in colon tissue and the intestinal inflammation is increased (Cheng et al., 2010). Treatment with the rodent-specific PXR ligand PCN protects against dextran sulfate sodium (DSS)-induced mouse acute colitis *via* suppressing NF-κB signaling pathway, but such treatment does not decrease the severity of DSS-induced colitis in PXR knockout mice, indicating a role for PXR agonist in protection against IBD (Shah et al., 2007). Our previous study indicated that natural flavonoid baicalein ameliorated DSS-induced colitis *via* a caudal type homeobox 2 (Cdx2)-mediated PXR activation mechanism (Dou et al., 2012a).

Over the past decades, the use of medicinal plants or their active components is becoming increasingly attractive options in the management of IBD (Yue et al., 2018). Alpinetin is an active flavonoid in plants of ginger family, especially in the seeds of *Alpinia katsumadai Hayata*. Aplinetin has been documented to possess multiple pharmacological activities including anti-bacterial, anti-inflammatory, and anti-tumor (Zhao et al., 2018). Several studies indicated that alpinetin downregulates the key molecules such as nitric oxide (NO), prostaglandin E2 (PGE2), tumor necrosis factor-alpha (TNF-α), and NF-κB during inflammatory states (Lee et al., 2012). Recently, alpinetin was shown to provide protection against DSS-induced colitis in mice *via* blockade of toll-like receptor-4 (TLR4)/NF-kB signaling pathway and NOD-like receptor protein 3 (NLRP3) inflammasome activation (He et al., 2016). Since inflammation is a hallmark of innate immunity, a potential role of PXR in regulating innate immunity through negative regulation of TLR4 was explored in recent years (Venkatesh et al., 2014). Our previous work demonstrated that alpinetin upregulates the expression of Cyp3a4 in LS174T human colorectal cells by activating the hPXR (Dou et al., 2012b). Thus, we hypothesized that the attenuated effects of alpinetin in experimental IBD might be associated with PXR regulation.

## Materials and Methods

### Cell Lines

HT-29 and LS174T human colon adenocarcinoma cell lines and RAW264.7 mouse macrophage cell line were obtained from the American Type Culture Collection (Manassas, VA). All cells were cultured in Dulbecco’s modified Eagle’s medium (DMED) supplemented with 10% fetal bovine serum (Life Technologies, NY) and a mixture of antibiotics (100 units/ml penicillin and 100 μg/ml streptomycin, Life Technologies) under 5% CO_2_ at 37°C.

### Mice

Healthy 8-week-old female C57BL/6 mice (20 ± 2 g) were obtained from the Shanghai Laboratory Animal Center. The subsequent experiments were performed in accordance with the guidelines approved by the Animal Ethics Committee of Shanghai University of Traditional Chinese Medicine (SHUTCM). All mice were housed under a specific pathogen-free facility and kept under the same conditions (25 ± 2°C, 60–70% humidity) with a 12 h light/dark cycle. Standard mouse food pellets and water were provided as required.

### Dextran Sulfate Sodium-Induced Colitis and Assessment of Colitis

**Figure 1:**
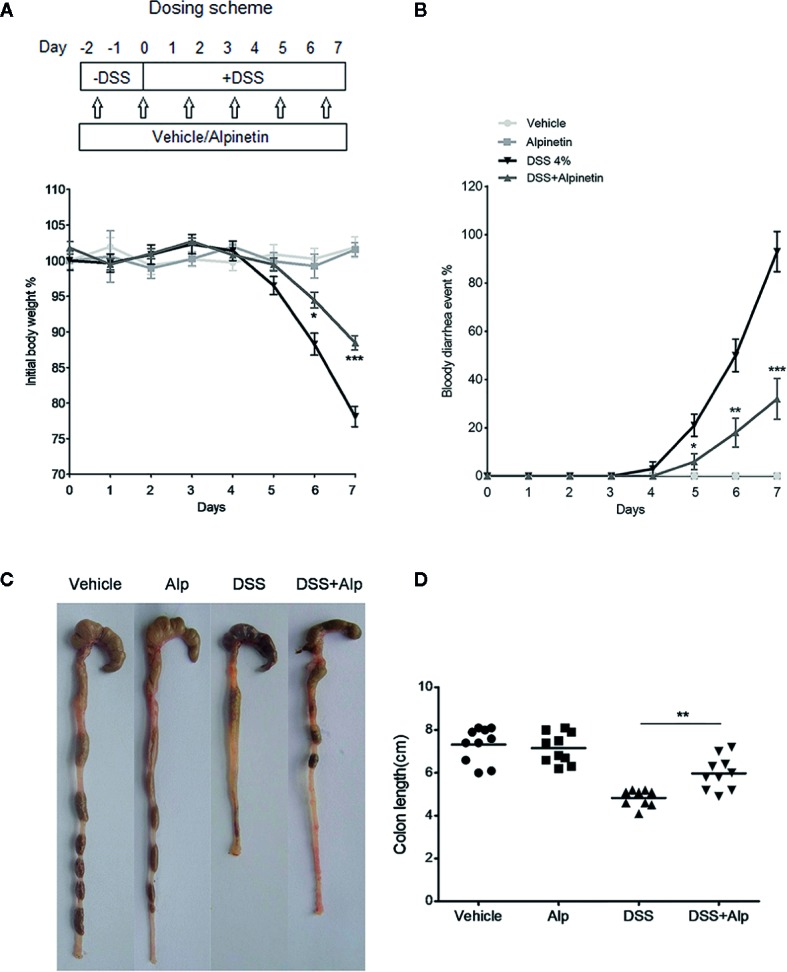
Alpinetin attenuated body weight loss, bloody diarrhea, and colon shortening in dextran sulfate sodium (DSS)-colitis mice. **(A)** Changes in body weight following DSS induction of colitis (left panel). The data are plotted as a percentage of the original body weight. The occurrence of bloody diarrhea (right panel). **(B)** The data are plotted as a percentage of the total mice that had bloody diarrhea at different time points of DSS treatment. **(C, D)** Macroscopic observation (left panel) and assessment of colon shortening (right panel) after DSS treatment. Data were expressed as the mean ± SD (n=10 per group). *p < 0.05, **p < 0.01, ***P< 0.001 *vs*. DSS-treated group.

Colitis was induced in mice by administering DSS in the drinking water *ad libitum* as described previously (Dou et al., 2012a). Four percent (w/v) DSS [molecular weight (MW) 36–50 kDa, MP Biomedicals, Solon, OH] was administered in the drinking water for 7 days, while control mice received tap water only. Alpinetin was dissolved in 0.5% methylcellulose and administered by oral gavage 2 days prior to DSS treatment and continued to the end of the DSS treatment (**Figure 1A**, upper panel). Mice were randomly divided into four groups (vehicle group, alpinetin group, DSS group, and DSS + Alpinetin group). Alpinetin (purity ≥ 98%, HPLC) was kindly provided by Shanghai R&D Center for Standardization of TCM (Shanghai, China). Alpinetin dosing (50 mg/kg per body weight) referred to previous report (He et al., 2016) and our preliminary studies.

Body weight, diarrhea, and bloody stool changes were monitored daily and mice were sacrificed under anesthesia after receiving the last gavage. Blood was collected, the spleen weight was recorded, and the colonic segments were placed on an ice-cold plate and cleaned of mesentery tissue and fat. The entire colon was removed, and the total length was measured. The distal colon was taken and immediately fixed in 10% formaldehyde for 24 h at room temperature, embedded in paraffin, and stained with hematoxylin and eosin (H&E) for histological evaluation. Histological injury was assessed as a combined score of inflammatory cell infiltration (score 0–3) and mucosal damage (score 0–3) using a previously described method (Zhang et al., 2014; Yue et al., 2018).

### Time-Resolved Fluorescence Resonance Energy Transfer Assay

The hPXR binding assay was performed using the LanthaScreen™ time-resolved fluorescence resonance energy transfer (TR-FRET) hPXR competitive binding assay system according to the manufacturer’s instructions (Life Technologies, NY), in which a test compound competes and displaces a reference fluorescent-labeled ligand from the recombinant terbium-labeled hPXR-LBD. Briefly, 10 μl of test compounds (0, 12.5, 25, 50, and 100 µM) was placed in quadruplicate into the wells of a black, round-bottomed 384-well assay plate. Next, 5 μl of 4× Fluormone hPXR Green was added to each well, followed by 5 μl of 4× hPXR-LBD (GST)/DTT/4×Tb anti-GST antibody. The plate was gently rocked and then incubated in the dark at room temperature for 1 h. TR-FRET was measured using an EnVision^®^ Multilabel Plate Reader (PerkinElmer, Boston, MA) at an excitation wavelength of 340 nm and at emission wavelengths of 520 and 495 nm. The TR-FRET ratio was calculated by dividing the emission signal at 520 nm by that at 495 nm. Rifampicin (10 µM, Sigma-Aldrich, St. Louis, MO) and SR12813 (1 µM, Sigma-Aldrich) was included as positive control of hPXR ligands.

### Gene Silencing

1×10^6^ LS174T cells were electroporated with the hPXR small-interfering RNA (siRNA) (sc-44057, Santa Cruz Biotech., CA) or the control siRNA using Lonza Nucleofector II instrument (Amaxa Biosystems, MD). The cells were then subjected to Western blot, quantitative PCR (qPCR), or NF-κB luciferase reporter assay.

### PXR-Mediated NF-κB Repression Reporter Assay

2×10^6^ LS174T cells in 100 μl transfection buffer (Cell Line Nucleofector Kit V) were co-electroporated with 1 μg pGL4.32[luc2P/NF-κB-RE/Hygro] luciferase reporter vector (Promega, Madison, WI), 0.5 μg expression vector (pSG5-hPXR or pSG5 control), and 0.1 μg pRL-TK vector using Lonza Nucleofector II instrument (program Q-009). For detailed plasmids information, please refer to our previous report (Dou et al., 2012a). The cells were transferred to 48-well plate following transfection and were treated with TNF-α (20 ng/ml, Sigma-Aldrich, St. Louis, MO) alone or in combination with alpinetin (25 μM) for 24 h. The cells were harvested in passive lysis buffer (Promega) and luciferase activity was detected using the dual-luciferase reporter assay system (Promega). Luminescence was detected by Turner Bio-systems Luminometer 20/20n (Turner Biosystems, CA). The results were expressed as the fold induction of the control cells.

### Determination of TNF-α and Interleukin-6 Levels

Colon segments were homogenized with ice-cold phosphate buffer saline (PBS). The homogenates were centrifuged at 3,000 *g* for 10 min. The levels of TNF-α and IL-6 in the supernatants were detected using mouse-specific enzyme-linked immunosorbent assay (ELISA) kits according to the manufacturer’s instructions (R&D systems, Minneapolis, MN) and the results were expressed as pg/mg of protein.

### Myeloperoxidase Assay

Myeloperoxidase (MPO) activity in colon tissue was measured using a detection kit according to the manufacturer’s instructions (Nanjing Jiancheng Bioengineering Institute, Nanjing, China) and the results were expressed as unit/g of tissue.

### Statistics

All data are expressed as the mean ± SD. The differences between groups were analyzed by one-way analysis of variance (ANOVA) followed by the least significant difference (LSD) for *post-hoc* test. Statistical analysis was performed by the SPSS 16.0 software package. P-values < 0.05 (two-sided) were considered significant.

## Results

### Alpinetin Attenuated Dextran Sulfate Sodium-Induced Mouse Colitis

**Figure 2:**
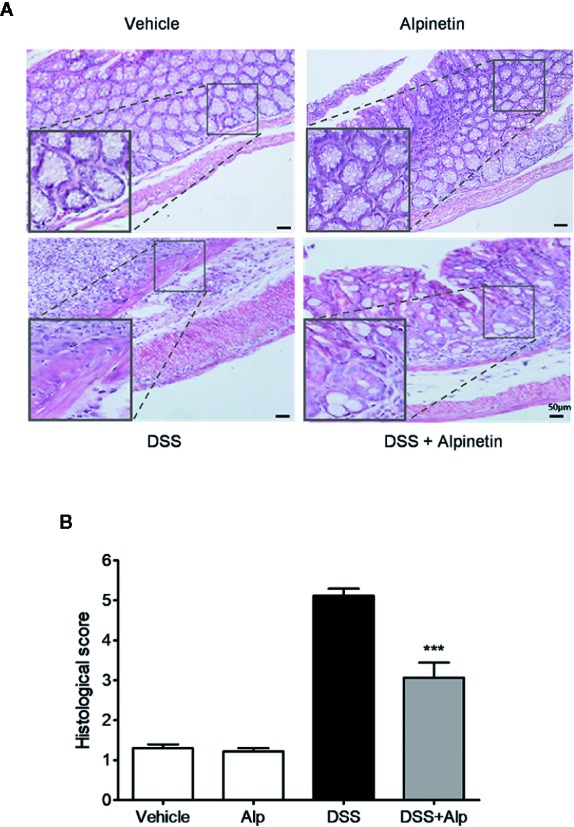
Alpinetin attenuated histopathologic injury in dextran sulfate sodium (DSS)-colitis mice. Representative H&E-stained colon sections **(A)** and histological score **(B)**. Scale bar corresponds to 50 μm and applies throughout. Data were expressed as mean ± SD (n=6 per group). ***P< 0.001 *vs*. DSS-treated group.

We added DSS to the drinking water to induce the acute colitis in mice. The inflammation was mainly localized to the colon, with features resembling human ulcerative colitis (UC). In mice receiving DSS treatment alone, a significant body weight loss (**Figure 1A**), bloody diarrhea (**Figure 1B**), colon shortening (**Figures 1C, D**), neutrophil infiltration, and histological damage was observed (**Figure 2**). Oral administration of alpinetin significantly ameliorated these hallmarks in DSS-colitis mice. In addition, none of the mice that received alpinetin alone exhibited loss of body weight, diarrhea, colon shortening, or mucosal disruption at any point during the study (**Figures 1** and **2**).

### Alpinetin Reduced the Activity of Myeloperoxidase and the Production of TNF-α and Interleukin-6 in Dextran Sulfate Sodium-Colitis Mice

**Figure 3:**
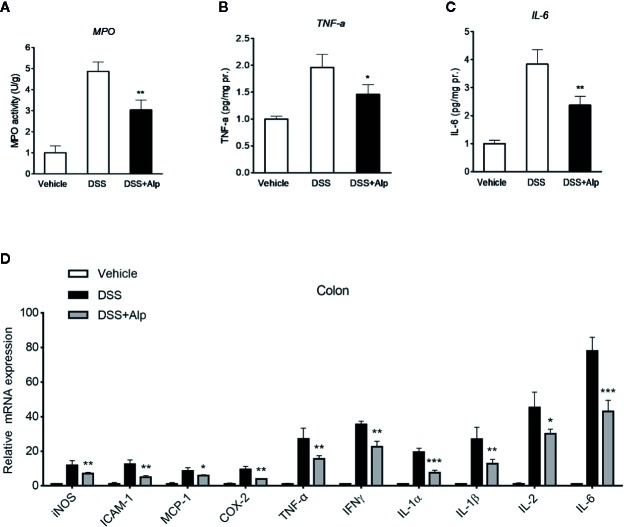
Alpinetin reduced the activity of myeloperoxidase (MPO), the production of TNF-α and interleukin (IL)-6, and the expression of NF-κB target genes in dextran sulfate sodium (DSS)-colitis mice. Colon segments from mice (n = 6 per group) were excised and homogenized. The supernatants were assayed for the determination of the activity of MPO **(A)** and the levels of TNF-α **(B)** and IL-6 **(C)** as described in the *Materials and Methods*. **(D)** Messenger RNA (mRNA) expression of inducible NO synthase (iNOS), ICAM-1, MCP-1, COX-2, TNF-α, IFNγ, IL-1α, IL-1β, and IL-6 in colon tissue was determined by quantitative real-time (qRT)-PCR. The expression level was normalized to β-actin. Values are expressed as mean ± SD (n=6). *p < 0.05, **p < 0.01, ***P< 0.001 *vs*. DSS-treated group.

Next, we investigated the activity of MPO, a marker for leukocyte infiltration into inflamed tissue (Zhang et al., 2014). Treatment of mice with DSS for 7 days notably increased the colon MPO activity (**Figure 3A**). However, the DSS-induced increase of MPO activity was significantly reduced after alpinetin treatment. In line with the MPO activity results, a significant increase in the content of TNF-α and IL-6 was observed in the colon of mice receiving 7 days DSS administration (**Figures 3B, C**). Alpinetin signiﬁcantly decreased the levels of TNF-α and IL-6 in the inflamed colon.

### Alpinetin Inhibited NF-κB Target Genes and Induced PXR Target Genes

**Figure 4:**
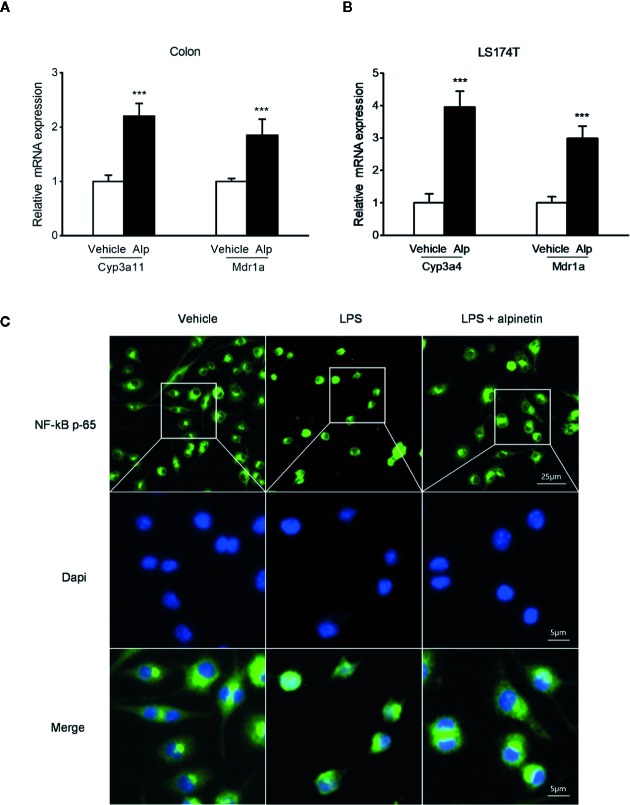
Alpinetin upregulated PXR target genes and inhibited NF-κB p-65 nuclear translocation. The messenger RNA (mRNA) expression of Cyp3a11 (Cyp3a4 human homolog) and Mdr1a was assessed by qRT-PCR in colon samples isolated from mice (n = 6 per group) treated with DSS alone or cotreated with alpinetin **(A)** or in LS174T cells treated with vehicle or 25 μM alpinetin for 24 h **(B)**. **(C)** NF-κB p65 nuclear translocation in RAW264.7 cells was evaluated by immunofluorescence staining and images were captured by a fluorescence microscope. Data were expressed as mean ± SD of three independent experiments. ***P < 0.001 *vs*. DSS-treated group or vehicle-treated cells.

In accord with the inhibition of MPO activity and cytokines production by alpinetin, a marked increase in the messenger RNA (mRNA) expression of NF-κB target genes was observed in the colon of mice that were exposed to DSS (**Figure 3D**). However, the increase in the expression levels of pro-inflammatory mediator genes following DSS administration was significantly decreased in mice receiving alpinetin treatment. Next, we sought to assess whether alpinetin affects PXR target genes expression. The mRNA expression of Cyp3a11 (Cyp3a4 human homolog) and multidrug resistance 1 (Mdr1) was determined. We observed a significant upregulation of the expression of Cyp3a11 and Mdr1a in the colon of mice that received DSS and alpinetin treatment compared with the mice receiving DSS alone treatment (**Figure 4A**). Consistent with the *in vivo* results, an increase in the mRNA expression of Cyp3a4 and Mdr1a following alpinetin treatment was observed in LS174T human colorectal cells, a cell line with abundant hPXR expression (**Figure 4B**).

### Alpinetin Inhibited NF-κB p-65 Nuclear Translocation in Raw264.7 Cells

We further evaluated whether alpinetin inhibited the nuclear translocation of p-p65 in RAW264.7 mouse macrophage cells, a widely used cell model for evaluating the *in vitro* anti-inflammatory effects of compounds (Dou et al., 2014a). As shown in **Figure 4C**, alpinetin obviously inhibited the nuclear translocation of p-p65 in lipopolysaccharide (LPS)-stimulated RAW267.4 cells.

### Alpinetin Inhibited NF-κB Activity in a PXR Dependent Manner

**Figure 5:**
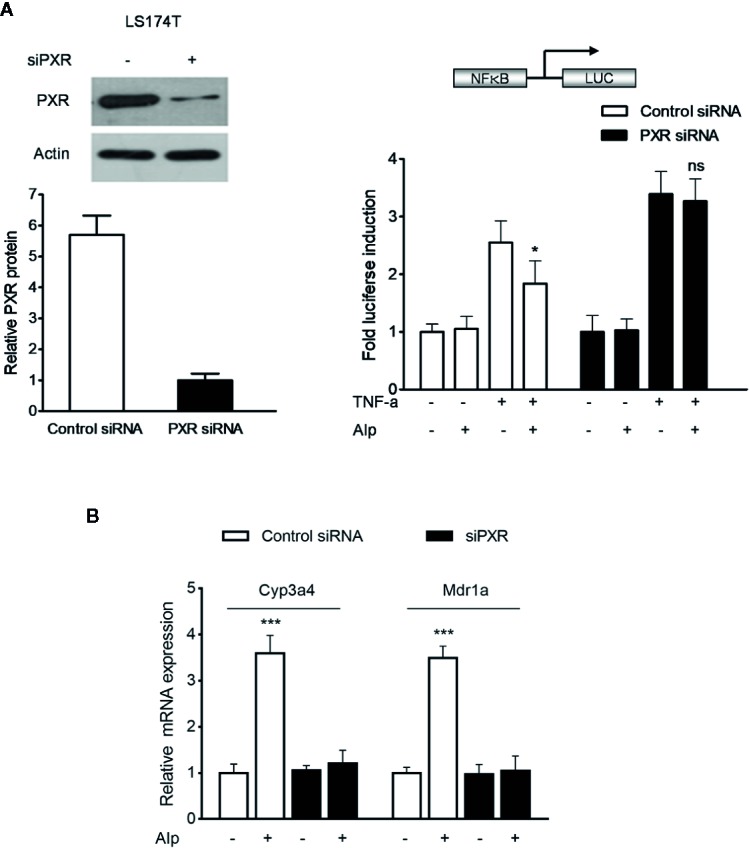
Alpinetin inhibited NF-κB activity and upregulated PXR target genes expression in a PXR dependent manner. The hPXR gene was silenced by PXR small-interfering RNA (siRNA) transfection in LS174T cells. The depletion of PXR was verified using western blot (**A**, left panel). The cells were then transfected with a pGL4.32[luc2P/NF-κB-RE/Hygro] reporter and incubated with TNF-α (20 ng/ml) alone or co-incubated with alpinetin (25 μM) for 24 h. A standard luciferase assay of the cell lysates was performed (**A**, right panel). **(B)** The mRNA expression of Cyp3a4 and Mdr1a in above PXR siRNA transfected LS174T cells was assessed using quantitative real-time (qRT)-PCR. The results are presented as the mean ± SD of three independent experiments. *p < 0.05, ***p < 0.001 *vs*. TNF-α-treated cells or vehicle-treated cells; ns, no significance.

To explore whether activation of PXR plays a critical role in alpinetin-mediated NF-κB repression, we transfected the hPXR siRNA into LS174T human colorectal cells, a cell line with abundant hPXR expression (Zhang et al., 2015a). As expected, knockdown of hPXR in LS174T cells by the hPXR siRNA significantly decreased the protein of hPXR (**Figure 5A**, left panel). The hPXR silenced LS174T cells were then transfected with a NF-κB-luciferase reporter and co-incubated with TNF-α and alpinetin. In cells transfected with the NF-κB-luciferase reporter and the control siRNA, treatment with the known NF-κB pathway activator TNF-α led to increased NF-κB activity, and the TNF-α-stimulated NF-κB activity was significantly inhibited by alpinetin (**Figure 5A**, right panel). However, in cells transfected with the hPXR siRNA, the regulatory effect of alpinetin on TNF-α-stimulated NF-κB activity was abolished.

### Alpinetin Upregulated PXR Target Genes Expression in a PXR Dependent Manner

Further, we sought to determine whether the alpinetin-induced upregulation of Cyp3a4 and Mdr1a was dependent on PXR activation. The hPXR gene expression in LS174T intestinal cells was silenced by hPXR siPXR transfection (**Figure 5A**, left panel). In control siRNA transfected cells, the mRNA expression of Cyp3a4 and Mdr1a were significantly upregulated after alpinetin treatment (**Figure 5B**). In contrast, the regulatory effect of alpinetin on the expression of Cyp3a4 and Mdr1a was abolished in cells transfected with hPXR siRNA.

### Alpinetin Activated Both Human and Mouse PXR

**Figure 6:**
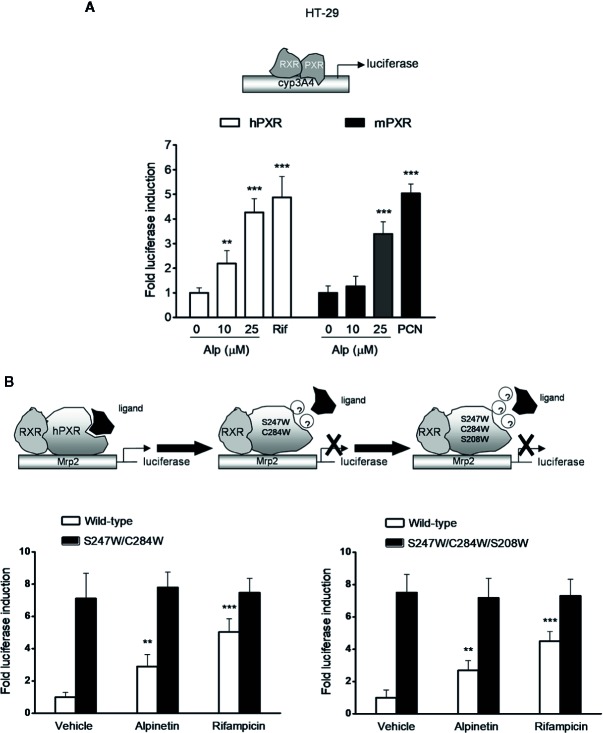
Alpinetin activated the wild-type hPXR but not the mutant hPXR. **(A)** HT-29 cells were co-transfected with CYP3A4-luciferase reporter combined with pRL-TK and the wild-type hPXR expression construct (pSG5-hPXR) or the wild-type mPXR expression construct (pSG5-mPXR). Cells were incubated with alpinetin (0, 10, and 25 μM) or rifampicin (10 μM) or PCN (5 μM) for 24 h. Cell extracts were assayed for luciferase activity. **(B)** HT-29 cells were co-transfected with Mrp2-luciferase reporter combined with pRL-TK and the wild-type hPXR expression construct (pSG5-hPXR) or the double-mutant (S247W/C284W) hPXR construct or the triple-mutant (S247W/C284W/S208W) hPXR construct. Cells were incubated with alpinetin (25 μM) or rifampicin (10 μM) for 24 h. Cell extracts were assayed for luciferase activity. The results were expressed as fold induction of the vehicle-treated cells. Data are presented as the mean ± SD of three independent experiments. **p < 0.01, ***p < 0.001 *vs*. vehicle-treated cells.

HT-29 colorectal cell line was used as a model for PXR transactivation assay because the expression level of PXR is low in this cell line (Ouyang et al., 2010). Using a transient hPXR transfection gene reporter assay, we observed that alpinetin, like rifampicin, dose-dependently activated the hPXR-driven Cyp3a4 promoter activity in HT-29 cells (**Figure 6A**). On the other hand, alpinetin, like the mPXR agonist PCN, dose-dependently induced the transactivation of the mPXR-driven Cyp3a4 promoter activity in HT-29 cells, indicating that alpinetin also activated mPXR (**Figure 6A**).

### Mutation of hPXR LBD Disrupted the Alpinetin-Induced hPXR Activation

To further evaluate whether alpinetin binds to the hPXR-LBD and subsequently activates the hPXR, we performed a transient transactivation reporter assay using the hPXR-LBD double-mutant (S247W/C284W) construct and the triple-mutant (S247W/C284W/S208W) construct, respectively. It has been reported that both the double-mutant and the triple-mutant effectively fill the pocket of the hPXR-LBD, leaving insufficient space for the established hPXR ligand SR12813 to bind to the receptor, which resulting in ligand-binding occlusion and ligand-independent constitutive activation (Wang et al., 2008; Venkatesh et al., 2011). We found that both alpinetin and rifampicin induced an increase in the wild-type hPXR-driven drug resistance protein 2 (Mrp2) promoter activity, whereas failed to activate either the double- or the triple-mutant hPXR-driven Mrp2 promoter activity (**Figure 6B**), indicating that the mutant hPXR-LBD disrupts the alpinetin-hPXR complex.

### Confirmation of the Interaction Between Alpinetin and the hPXR-LBD

**Figure 7:**
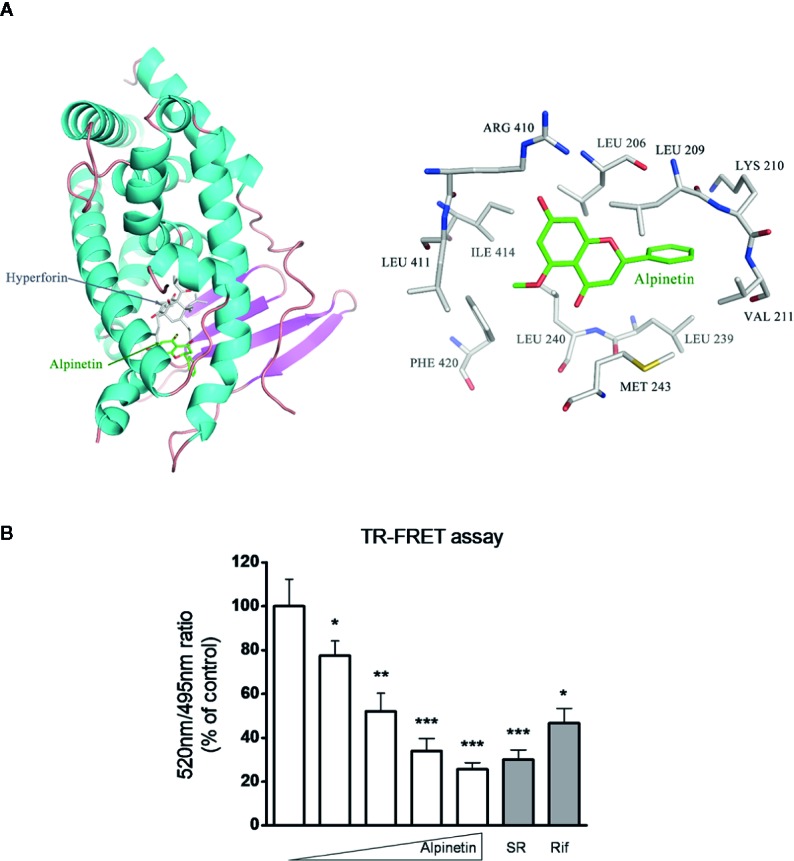
Alpinetin bound to the hPXR-LBD was confirmed by TR-FRET assay. **(A)** Molecular docking analysis was performed to investigate the potential combination mechanism of alpinetin binding to hPXR protein. **(B)** A LanthaScreen TR-FRET competitive binding assay was performed to evaluate the interaction between alpinetin (0, 5, 10, 25, and 50 µM) and the hPXR-LBD. Rifampicin (10 µM) and SR12813 (1 µM) were included as positive control compounds. The TR-FRET ratio was calculated by dividing the emission signal at 520 nm by that at 495 nm. Data are expressed at means ± SD of three independent experiments. *P < 0.05, **P < 0.01, ***P < 0.001 *vs*. vehicle-treated wells.

In the current study, molecular docking analysis was performed to investigate the potential combination mechanism of alpinetin binding to hPXR protein. As was shown in **Figure 7A**, alpinetin was fitted into the hydrophobic pocket of hPXR-LBD. To confirm the direct binding of alpinetin to the hPXR-LBD, a TR-FRET competitive hPXR-LBD binding assay was performed. The results showed that alpinetin concentration-dependently decreased the TR-FRET emission ratio (**Figure 7B**). As positive controls, both rifampicin and SR12813 significantly decreased the TR-FRET emission ratio as predicted.

## Discussion

Chemically induced colitis models have been developed and extensively used to elucidate the pathogenic mechanisms of IBD. One of the most widely used experimental models is developed by treating animals with DSS in drinking water for 6–10 days. The resulting inflammation generally affects the mucosal lining of the intestinal wall, with disease features that resemble human UC (Neurath and Travis, 2012). In the present study, we found that alpinetin attenuated the DSS-induced weight loss, bloody diarrhea, colon shortening, inflammatory infiltration, and histological injury in mice. Notably, none of the mice that received alpinetin alone exhibited apparent body weight loss, diarrhea, colon shortening, or mucosal disruption throughout the study, indicating a relative safety of alpinetin treatment.

As a member of the nuclear receptors family of ligand-activated transcription factors, PXR is predominantly expressed in the liver and gastrointestinal tract and has been characterized as a xenobiotic sensor that is activated by structurally diverse chemicals (Mani et al., 2013). Activation of PXR promotes the expression of xenobiotic oxidation and conjugation enzymes and transporters involved in the elimination of harmful chemicals and organisms from the body (Dou et al., 2014b). Cyp3a4 (Cyp3a11 rodent homolog) and Mdr1, both abundantly expressed in the mammalian liver and small intestine, are xenobiotic metabolizing enzymes and PXR target genes. Induction of Cyp3a4 and Mdr1 contributes to the metabolism of approximately 50% of clinically used drugs and a large amount of xenobiotics (Mani et al., 2013). Gene expression analysis has indicated significant downregulation of PXR and its target genes in the colon of IBD patients (Zhang X. et al., 2015). Downregulation of Cyp3a and Mdr1 was also observed in the intestine of DSS-treated mice (Kawauchi et al., 2014). Similar to previous reports, we found that DSS decreased the levels of Cyp3a11 and Mdr1a in the colon, but alpinetin reversed the DSS-mediated downregulation of Cyp3a11 and Mdr1a*. In vitro,* alpinetin increased the expression of Cyp3a4 and Mdr1 in LS174T human colorectal cells; however, knock down of PXR abolished the induction for these genes, indicating that PXR is critically involved as a mediator. It has been suggested that the detoxification properties of PXR and its target genes are necessary to maintain the integrity of the intestinal epithelial barrier, which has beneficial effects for intestinal inflammation (Zhang et al., 2015b). Thus, the PXR-mediated upregulation of xenobiotic detoxification genes might contribute to the attenuated effects of alpinetin in experimental IBD.

It has been documented that activation of PXR suppresses the NF-κB signaling molecules in the colon of DSS colitis mice (Shah et al., 2007). Cheng et al. reported that treatment of PXR-humanized mice with rifaximin, a human-specific PXR agonist, results in significant inhibition of NF-κB and its target genes in the intestine in experimental mouse model of DSS coliti, suggesting a potential role for PXR agonists in abrogating IBD (Cheng et al., 2010). More recently, the same group reported that rifaximin compromises NF-κB signal and elicits protection in a PXR-humanized mouse model of colitis-associated cancer, which is associated with reduced inflammation, cancer cell proliferation, and pro-apoptosis (Cheng et al., 2014). In the current study, we found that alpinetin decreased the activation of NF-κB, the expression of pro-inflammatory mediator genes, the activity of MPO, and the production of TNF-α and IL-6 in DSS colitis mice. In addition, alpinetin blocked the nuclear translocation of p-p65 in LPS-stimulated RAW264.7 macrophages. Interestingly, silencing hPXR gene by siRNA demonstrated the necessity for hPXR in alpinetin-mediated inhibition of NF-κB activation in LS174T cells. Further studies disclosed that alpinetin activated both the mouse and the human PXR; however, alpinetin could not activate either the double-mutant (S247W/C284W) or the triple-mutant (S247W/C284W/S208W) within the LBD area of hPXR. The competitive hPXR ligand binding assay confirmed the binding interaction between the hPXR-LBD and alpinetin. Collectively, these findings suggest that alpinetin appears to exert its amelioration of DSS-induced colitis through PXR-mediated NF-κB inhibition.

In conclusion, the current study demonstrated that alpinetin behaved as a PXR ligand and decreased the susceptibility of mice to DSS-induced colitis *via* a mechanism associated with PXR-mediated anti-inflammation and detoxification. The present study may provide insight into the potential use of alpinetin in the treatment of human IBD.

## Data Availability Statement 

All datasets generated for this study are included in the article/**Supplementary Material**.

## Ethics Statement

The animal study was reviewed and approved by Animal Ethics Committee of Shanghai University of Traditional Chinese Medicine (SHUTCM).

## Author Contributions

ZY, BY, and LD performed the animal experiments and the cell-based assays. XL, YR and JZ performed the biochemical analysis. XL and BY analyzed the data. ZW contributed reagents or analytic materials. SM and WD participated in research design and manuscript writing.

## Funding

This work was supported by National Natural Science Foundation of China (81530096, U1032604), Natural Science Foundation of Shanghai (17ZR1427800) and National Institutes of Health Grant (R01 CA2222469).

## Conflict of Interest

The authors declare that the research was conducted in the absence of any commercial or financial relationships that could be construed as a potential conflict of interest.

## Abbreviations

Alp, alpinetin; Cdx2, caudal type homeobox 2; COX-2, cyclooxygenase 2; Cyps, cytochromes; DMEM, Dulbecco’s modified Eagle’s medium; DSS, dextran sulfate sodium; ECL, enhanced chemiluminescence; ELISA, enzyme-linked immunosorbent assay; H&E, hematoxylin and eosin; IBD, inflammatory bowel disease; ICAM-1, intercellular adhesion molecule-1; IFNγ, interferon-gamma; IL-1α/1ß/2/6, interleukin 1α/1ß/6; iNOS, inducible NO synthase; LBD, ligand-binding domain; LPS, lipopolysaccharide; LSD, least significant difference; MCP-1, monocyte chemotactic protein-1; MPO, myeloperoxidase; Mrp2, drug resistance protein 2; NF-κB, nuclear factor-kappa B; NLRP3, NOD-like receptor protein 3; NO, nitric oxide; PBS, phosphate buffer saline; PCN, pregnenolone-16α-carbonitrile; PGE2, prostaglandin E2; PXR, pregnane X receptor; qPCR, quantitative polymerase chain reaction; RXRα, retinoid X receptor alpha; SHUTCM, Shanghai University of Traditional Chinese Medicine; TCM, traditional Chinese medicine; TLR4, toll-like receptor-4; TNF-α, tumor necrosis factor-α; TR-FRET, time-resolved fluorescence resonance energy transfer; UC, ulcerative colitis.
