# Supplementary material for: Activation of PXR by Alpinetin Contributes to Abrogate Chemically Induced Inflammatory Bowel Disease
Source: Front Pharmacol. 2020 Apr 21;11:474. doi: 10.3389/fphar.2020.00474 (PMC7186371; doi:10.3389/fphar.2020.00474)
Supplement: Supplementary file 1 [file DataSheet_1.doc]

[**Activation of PXR by alpinetin contributes to abrogate chemically induced inflammatory bowel disease**](https://www.ncbi.nlm.nih.gov/pubmed/27611972)

Zhilun Yu^1#^, Bei Yue^1#^, Lili Ding^1#^, Xiaoping Luo^1^, Yijing Ren^1^, Jingjing Zhang^1^, Sridhar Mani^2^,, Zhengtao Wang^1*^ and Wei Dou^1*^

This file includes: Supplemental Methods, including real-time quantitative polymerase chain reaction (qPCR), western blot analysis, immunofluorescence staining, wile-type/mutant PXR transactivation reporter assay, and *in silico* docking analysis.

**Supplemental Methods**

**Western blot analysis**

Cells in RIPA lysis buffer (Thermo Scientific, Waltham, MA) supplemented with fresh protease inhibitor and phosphatase cocktail tablets were disrupted by incubation for 30 minutes on ice or by homogenization on ice, respectively. The lysates were centrifuged at 4°C (12,000 *g*, 15 min) and the supernatants were collected. Equal amounts of protein (30 µg) were separated by 10% SDS-PAGE and transferred to nitrocellulose membranes (Thermo Scientific). Membranes were blocked in 5% (w/v) skim milk and immunoblotted with the antibodies against the hPXR (Cell Signaling Technology, Danvers, MA) and β-actin (Santa Cruz Biotechnology, Dallas, TX). The membranes were incubated with appropriate secondary antibodies (Santa Cruz) and developed by enhanced chemiluminescence (ECL) western blotting detection reagents (Thermo Scientific). The protein bands were analyzed using a GS-700 imaging densitometer (Bio-Rad, CA) and quantified following normalization to the expression of β-actin.

**RNA analysis**

Total RNA from colon samples or cultured cells was extracted using TRIzol reagent (Thermo Scientific) according to the manufacturer’s instructions. cDNA was synthesized from 3 μg of total RNA using the SuperScript II Reverse Transcriptase kit (Life Technologies, Carlsbad, CA). Quantitative real-time polymerase chain reaction (qPCR) was carried out using SYBR Premix ExTaq Mix (Takara Bio Inc., Otsu, Japan) and quantitatively measured with an ABI Prism 7900HT Sequence Detection System (Life Technologies). Results were normalized as the ratio of optimal density relative to β-actin. Primer sequences are as follows (forward 5’-3’, reverse 5’-3’): 5’-GATGAAAGAAAGTCGCCTCG-3’/5’-GCTGGACATCAGGGTGAGTG-3’ for hCyp3a4, 5’-AGCCCATCCTGTTTGACTGC-3’/5’-TGTATGTTGGCCTCCTTTGC-3’ for hMdr1a, 5’-GGAAATCGTGCGTGACATTA-3’/5’-TCAGGCAGCTCGTAGCTCTT-3’ for hß-actin, 5’-TGGAGATGGAATACCTGGAT-3’/5’-GAATCATCACTGTTGACCCT-3’ for mCyp3a11, 5’-TGTGATTGCGTTTGGAGGAC-3’/5’-CCATACCAGAATGCCAGAGC-3’ for mMdr1a, 5’-GGGAATCTTGGAGCGAGTTG-3’/5’-GTGAGGGCTTGGCTGAGTGA-3’ for miNOS, 5’-CGCTGTGCTTTGAGAACTGT-3’/5’-AGGTCCTTGCCTACTTGCTG-3’ for mICAM-1, 5’-AAGTTGACCCGTAAATCTGA-3’/5’-TGAAAGGGAATACCATAACA-3’ for mMCP-1, 5’-GAAGTCTTTGGTCTGGTGCCT-3’/5’-GCTCCTGCTTGAGTATGTCG-3’ for mCOX-2, 5’-CGTGGAACTGGCAGAAGAGG-3’/5’-AGACAGAAGAGCGTGGTGGC-3’ for mTNF-α,

5’-AGCAACAACATAAGCGTCAT-3’/5’-CCTCAAACTTGGCAATACTC-3’ for mIFNr, 5’-GTTCTGCCATTGACCATCTC-3’/5’-TGATACTGTCACCCGGCTCT-3’ for mIL-1α, 5’-GGCTGGACTGTTTCTAATGC-3’/5’-ATGGTTTCTTGTGACCCTGA-3’ for mIL-1ß, 5’-TCAGCAACTGTGGTGGACTT-3’/5’-AGTGATTAGCAAGGGTGAGA-3’ for mIL-2, 5’-ATGGCAATTCTGATTGTATG-3’/5’-GACTCTGGCTTTGTCTTTCT-3’ for mIL-6, and 5’-CAGCCTTCCTTCTTGGGTAT-3’/5’-TGGCATAGAGGTCTTTACGG-3’ for mß-actin. PCR reactions were carried out using SYBR Premix ExTaq Mix (Takara, Japan) in an ABI Prism 7900 real-time PCR System (Life Technologies). The thermal cycler parameters were as follows: 1 cycle of 95°C for 30 s, then 40 cycles of denaturation (95°C, 5 s) and combined annealing/extension (60°C, 30 s). Gene expression changes were calculated by the comparative Ct method, and the values were normalized to the internal ß-actin control.

**Immunofluorescence staining**

The immunostaing was performed as described previously (Zhang et al., 2014). Briefly, RAW264.7 cells were seeded on sterile coverslips with a density of 5 × 10^4^/well in a 24-well plate for 24 hours until cell attachment. The cells were pretreated with alpinetin (25 µM) for 2 h following stimulation with LPS (2 μg/ml) for 12 h. The cells were fixed in 4% (w/v) paraformaldehyde for 10 min at room temperature, and washed three times with PBS, then permeabilized with 0.3 % (w/v) Triton X-100 (Sigma-Aldrich, St. Louis, MO) for 20 min at room temperature, followed by three washes with PBS. After incubation in PBS containing 10% bovine serum albumin (Sigma-Aldrich) for 30 min at room temperature, the slides were incubated with antibody against p-p65-NLS (Thermo Scientific) overnight at 4 °C and then incubated with Alexa Fluor 488-conjugated secondary antibody (Thermo Scientific) for 1 h in the dark. To stain the nuclei, 1 µg/ml of 4′,6-diamidino-2-phenylindole (Sigma-Aldrich) in PBS was added before capturing images with a ﬂuorescence microscope (Olympus CKX41, Tokyo, Japan).

**Wild-type/mutant PXR transactivation reporter assay**

For the wild-type PXR transactivation assay, 1×10^6^ HT-29 cells were transfected with 1 μg CYP3A4-luciferase reporter combined with 0.1 μg pRL-TK, and 0.5 μg of plasmid expressing wild-type human PXR (pSG5-hPXR) or wild-type mouse PXR (pSG5-mPXR) using Lonza Nucleofector II instrument (Amaxa Biosystems, MD). For the mutant PXR transactivation assay, the cells were transfected with 1 μg CYP3A4-luciferase reporter combined with 0.1 μg pRL-TK, and 0.5 μg plasmid expressing the wild-type hPXR or the double-mutant (S247W/C284W) or the triple-mutant (S247W/C284W/S208W) hPXR, as shown in the schematics (Fig. 6B). For detailed plasmid information, please refer to our previous reports (Wang et al., 2008; Venkatesh et al., 2011). The cells were incubated with alpinetin (0, 10 and 25 μM) or rifampicin (10 μM) or PCN (5 μM) for 24 h and were harvested in passive lysis buffer (Promega, Madison, WI). The luciferase activity was detected using the dual-luciferase reporter assay system (Promega). The results are expressed as the fold induction of control cells.

***In silico* docking analysis**

*The in silico* modeling study of the hPXR ligand affinity was performed as described previously (Dou et al., 2012). The hPXR ligand hyperforin (Sigma-Aldrich) was served as a template molecule to evaluate the ligand affinity. Three-dimensional structure of hPXR co-crystalized with hyperforin was obtained from Protein Data Bank (PDB code: 1M13). The co-crystalized structure was constructed using the MOE (Molecular Operating Environment, version 2012.10, Chemical Computing Group, Montreal, Canada) program. The binding site has been well characterized based on structural information derived from a variety of co-crystals (PDB Code: 1ILH/1M13/1SKX/2QNV/3R8D). PLIF (Protein Ligand Interaction Fingerprints) program was used to analyze the co-crystals and identify the conserved pocket residues. The constructed structure was submitted to FlexX (BioSolveIT, Germany) procedure for docking analysis. The residues around hyperforin (template ligand) within 7Å were selected as the binding groups, which includes all the critical residues. Then, hyperforin was removed and alpinetin was docked into the crystal structure. The docking mode was analyzed by MOE program following the energy minimization.

**References**

1. Zhang, J., Dou, W., Zhang, E., Sun, A., Ding, L., Wei, X., et al. (2014). Paeoniflorin abrogates DSS-induced colitis via a TLR4-dependent pathway. Am. J. Physiol. Gastrointest. Liver. Physiol. 306(1):G27-36. doi: 10.1152/ajpgi.00465.2012.
2. Wang, H., Li, H., Moore, L.B., Johnson, M.D., Maglich, J.M., Goodwin B, et al. (2008). The phytoestrogen coumestrol is a naturally occurring antagonist of the human pregnane X receptor. Mol. Endocrinol. 22:838–57. doi: [10.1210/me.2007-0218](https://doi.org/10.1210/me.2007-0218)
3. [Venkatesh, M](https://www.ncbi.nlm.nih.gov/pubmed/?term=Venkatesh M[Author]&cauthor=true&cauthor_uid=21464197)., [Wang, H](https://www.ncbi.nlm.nih.gov/pubmed/?term=Wang H[Author]&cauthor=true&cauthor_uid=21464197)., [Cayer, J](https://www.ncbi.nlm.nih.gov/pubmed/?term=Cayer J[Author]&cauthor=true&cauthor_uid=21464197)., [Leroux, M](https://www.ncbi.nlm.nih.gov/pubmed/?term=Leroux M[Author]&cauthor=true&cauthor_uid=21464197)., [Salvail, D](https://www.ncbi.nlm.nih.gov/pubmed/?term=Salvail D[Author]&cauthor=true&cauthor_uid=21464197)., [Das, B](https://www.ncbi.nlm.nih.gov/pubmed/?term=Das B[Author]&cauthor=true&cauthor_uid=21464197)., et al. (2011). In vivo and in vitro characterization of a first-in-class novel azole analog that targets pregnane X receptor activation. Mol. Pharmacol. 80:124–35. doi: 10.1124/mol.111.071787.
4. Dou, W., Mukherjee, S., Li, H., Venkatesh, M., Wang, H., Kortagere, S., et al. (2012). [Alleviation of gut inflammation by Cdx2/Pxr pathway in a mouse model of chemical colitis.](https://www.ncbi.nlm.nih.gov/pubmed/22815676) PLoS. One. 7(7):e36075. doi: 10.1371/journal.pone.0036075.
